# Assessment of oral health knowledge, literacy, and attitude among schoolteachers towards oral health - A cross-sectional study

**DOI:** 10.1186/s12903-023-03012-z

**Published:** 2023-06-14

**Authors:** Omir Aldowah, Ali A Assiry, Nizar F Mujallid, Farid N Ashi, Faisal Abduljawad, Minna M. Al-Zahrani, Rawam Ezzaddin, Mohmed Isaqali Karobari

**Affiliations:** 1 Faculty of Dentistry, Najran University, Kingdom of Saudi Arabia, Najran, 11001 Kingdom of Saudi Arabia; 2 Pediatric Dentistry Department, King Fahad General Hospital, Jeddah, Kingdom of Saudi Arabia; 3 Resident Dentist, Ministry of Health, Jeddah region, Jeddah, Kingdom of Saudi Arabia; 4 Resident Dentist, BDC, Ministry of Health, Jeddah region, Jeddah, Kingdom of Saudi Arabia; 5 BDS, Ibn Sina College, Jeddah, Kingdom of Saudi Arabia; 6 Department of Conservative Dentistry & Endodontics, Faculty of Dentistry, University of Puthisastra, Phnom Penh, 12211 Cambodia; 7 Center for Transdisciplinary Research, Saveetha Institute of Medical and Technical Science, Saveetha Dental College, Saveetha University, Chennai, India

**Keywords:** Dental, Oral health literacy, Schoolteachers, Knowledge, Attitude

## Abstract

**Background:**

To accomplish the purpose of health education and health promotion programs, schools are the most effective place for delivering health information to children. The main purpose of our research was to inform, gather evidence and contribute to developing knowledge regarding the OHL, knowledge, and attitude among school teachers towards oral health in Najran region of Saudi Arabia.

**Methods:**

A questionnaire-based cross-sectional study was conducted in Najran region of Saudi Arabia for 6 months. A stratified cluster random sampling of 252 teachers was taken to represent all the teachers of Najran region of Saudi Arabia. The questionnaire contains 2 parts, sociodemographic part that include age, gender, education, teaching level, and income of the participants. The second part contains 25 items that assess the OHL (HelD-14 questions), knowledge (6 questions), and attitude (5 questions) of the participants. SPSS software version 26 was used to enter and analyze the data (IBM SPSS, Chicago, IL, USA software version 26.0). Multiple Logistic regression was applied to check the relationship between OHL and the associated factors. The Chi-square test was applied to evaluate knowledge of study participants. The level of significance was set up at p < 0.05.

**Results:**

A total of 252 school teachers with a Mean age of school teachers 32.25 ± 8.46 participated in the study. The multiple logistic regression model shows the association between age, education, and OHL level of school teachers. After adjustment for sociodemographic factors age (OR = 0.219, 95% CI: 0.058–0.834), education (OR = 9.053, 95% CI: 1.135–72,023) were significantly associated with OHL of school teachers. Female participants showed better performance with respect to all the knowledge questions, a significantly higher level of knowledge (p-value < 0.05) was reported with all the questions except the second question (dental plaques causes). 94.8% of teachers agreed that children’s teeth should be checked by a dentist on a regular basis, while 96.8% agreed that dental health education should be included in the primary school curriculum and that all teachers should receive dental health education training.

**Conclusion:**

Overall, school teachers have high oral health literacy, adequate knowledge, and a positive attitude toward oral health. The female teachers had more knowledge about dentistry than their male counterparts.

## Introduction

Oral health literacy refers to an individual’s ability to acquire, process, and comprehend basic oral health - related information and the services required to make sensible oral health choices [1]. A familiarity, understanding, or awareness of something, such as details, abilities, or objects, is knowledge. A person’s attitude is a combination of values, thoughts, and feelings that predispose them to react to things, individuals, processes, or institutions positively or negatively [2]. Children spend a significant amount of time in the schools [3]. Knowledge is effectively passed on to students by their teachers, who also play an important role in molding the students’ behaviour and attitudes. They have the potential to educate the next generation of people who will be using health care services and become knowledgeable consumers [4]. Teachers are considered as role models for communicating life values [5]. Because of their experience, school teachers have the potential to affect a significant number of children and therefore play an essential role in the preparation and accomplishment of oral health prevention programs. In addition, children can be approached at a time when their health patterns are developing [6], and oral health services can be made accessible to all children, including those who may not have access to other sources of health information, such as the dental clinic [7]. The school teachers are an invaluable asset in the fight against avoidable diseases like oral disorders because of the impact they may have on vast populations of children and their parents [8]. Schools have a vast capacity to promote services for children that include preventive health and preventive dentistry [9].

The school teachers’ oral health knowledge is essential for their oral health and the children’s oral health with whom they communicate and teach [10]. Knowledge about oral health is known as a prerequisite for activities related to health [11]. Multiple studies have reported that better oral health knowledge is helpful for the prevention of dental diseases and improved oral health status [12, 13]. A previous study on school teachers’ knowledge of preventive dentistry discovered that the school teachers were misinformed about the specifics of preventive dentistry [14]. The several benefits of using school teachers for health promotion and health education activities include consistency of teaching, incorporating oral and general health with other activities, and low costs associated with such services [15, 16]. Barriers to teaching dental health education in schools have been described as a shortage of sufficient oral health knowledge, literacy, attitude, and training on aspects of oral health, time, deficiency of funding, and a failure to integrate oral health into the school curriculum [17, 18]. Since school teachers can play a key role in disseminating preventive information and health promotion, their own OHL, attitude, and knowledge must represent professional advice.

The World Health Organization (WHO) initiated a Global School Health Initiative in 1995, emphasizing the role of schools in providing health education to schoolchildren [19]. It has been noted that many school teachers provide inadequate or even erroneous information about oral health to their students [20]. According to the findings of a study that analyzed teachers’ knowledge of preventive dentistry, it was determined that teachers in primary schools lacked adequate knowledge of the specifics of preventive dentistry [9, 14]. The present study is conducted in Saudi Arabia, which is a significant nation located in the Middle East. The nation is divided into 13 regions, and its landmass encompasses approximately 2.15 million square kilometers, giving it a massive proportion of the Arabian Peninsula [21]. According to a recent study conducted in the Kingdom of Saudi Arabia, 73% of 120 school teachers were aware of the important role of sugar and bacteria in the advancement of dental caries, 68% are aware of bleeding gums with inappropriate tooth cleaning, and 76% believed that tooth brushing could help stop dental caries. Furthermore, 56% agreed that school teachers should teach children about dental disease causes [16]. Another study conducted by Khan et al. in Riyadh City, Saudi Arabia, discovered that 34% of school teachers had good dental hygiene, 50.2% had fair oral hygiene, and 15.8% had poor oral hygiene. In terms of knowledge, 65% of school teachers knew about tooth decay, while 45% knew about gum diseases [22]. Schools can serve as an essential platform for increasing students’ general and oral health knowledge. Through their educational and daily interaction with students, teachers will play a positive role in helping students maintain good oral health by encouraging oral hygiene [23]. In order to reap the benefits of having OHL abilities later in life, the seeds of those skills should be planted early in life. In order to accomplish this goal, OHL should be taught alongside general literacy during their time spent in school. The students will carry with them throughout their entire lives the knowledge and abilities that they gained while they were attending school. In most cases, the students imitate the actions and words of their teachers [24]. Therefore, the teachers play a significant part in the development and administration of preventative programmes related to oral health. Because of the high prevalence of oral diseases and the lack of information regarding the efficacy of school-based oral health programmes in Saudi Arabia [25], The findings of this study will be helpful in understanding the level of preparedness of school teachers to administer a coordinated school oral health program to overcome the potential barriers. Therefore, the main purpose of our research was to inform, gather evidence and contribute to developing knowledge regarding the OHL, knowledge, and attitude among school teachers towards oral health in Najran region of Saudi Arabia.

## Methodology

Ethical clearance was obtained to conduct the study from Najran University with the ethical number 2001/00031/2.

### Study design and participants

**Fig. 1:**
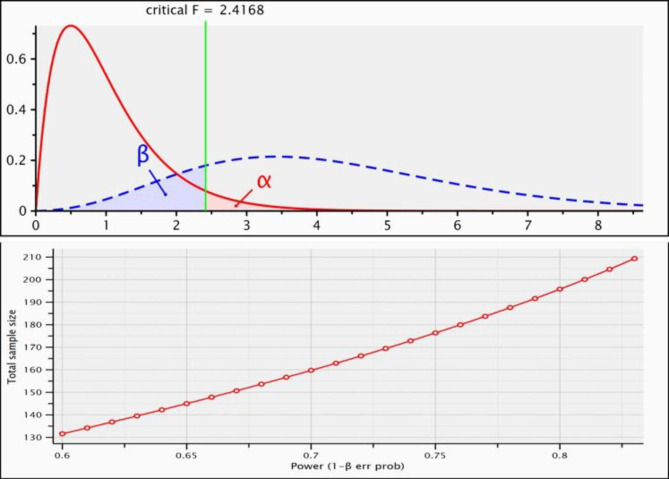
Sample size calculation plots

This was a cross-sectional questionnaire-based study done among the Saudi population living in Najran city of Saudi Arabia for a period of 6 months. The current study was carried out in state schools across all three levels of education ( primary, middle, and secondary) in the city of Najran, which is situated in the southwestern part of the country and has a total population of 386,750 people. There are 476 public schools in Najran, 240 of which are designated as schools for boys, and 236 of which are designated as schools for females [26]. Using stratified cluster random sampling, a total of 252 school teachers were randomly selected for the study. Before the commencement of the study, permissions were obtained by sending letters to the schools and concerned authorities explaining the objective of our study. The school teachers were fully informed about the purpose, method, and possible uses of the research data. Consent was obtained before the data collection from the school teachers to participate voluntarily in the research. Those who met the inclusion criteria were the teachers who teach in primary, middle, and secondary schools working in private and government schools as well as those who serve as principals or head masters in those levels. Participants who did not provide informed consent were excluded from the study. The participants were given the right to leave the research during the data collection. The sample size calculation was done using G Power 3.1.9.5 software for sample size, using F-test, Anova: Fixed effects, omnibus, one-way. The type of power analysis was A priori: compute required sample size-given α, power, and effect size. The total requires sample size obtained was 205 (Fig. 1). Following the 20% dropout due to non-response, incomplete data and loss-to-follow up, a total of 252 participants were included in the study.

### Questionnaire design and validation

Before the commencement of the study, the questionnaire was designed based on information from earlier studies. The questionnaire was first translated into Arabic using standard methods. At the first stage, the questionnaire was separately translated into Arabic by two translators with expertise in translating technical texts from English to Arabic. The translated questionnaires were then returned to English by two translators who had experience translating from Arabic into English but were unaware of the original English text of the questionnaire. The two English versions of the questionnaires (translated and original) were then compared, and if there was any disagreement, a session with the expert committee was convened to obtain an agreement on the final translated text. The reliability of questionnaire has been validated by authorities in the field of dentistry. Cronbach’s alpha was checked in SPSS version 26.0 (IBM SPSS, Chicago, IL,USA) to examine the questionnaire’s internal consistency. A value of 0.760 was obtained which is regarded as an acceptable level of reliability for research instruments. The questionnaire’s initial version was put through pilot testing to ensure its validity and usability. Feedback from respondents on the questionnaire’s format and wording was helpful in making it more effective. The questionnaire was pretested on 10% of the total sample size.

### Data collection

The selected teachers were provided with a brief explanation of the study. Teachers were given self-reported questionnaire by the appointed research assistant consisting of 25 questions including personal information, OHL, knowledge, and attitudes. The questionnaire contains 2 parts, sociodemographic part that include age, gender, education, teaching level, and income of the participants. The second part contains 25 items that assess the OHL (14 questions) [27], knowledge (6 questions) [28], and attitude (5 questions) [8] of the participants. The participants were instructed to answer each item using the response format given at the end of the questionnaire. The research assistant received training that focused on the theoretical components of the questionnaire. The questionnaire was distributed to the teachers at the school, and they were given a period of around half an hour to fill out the form. It was recommended that any teachers who struggled to comprehend any of the terminology or comments included in the questionnaire seek assistance from an experienced research assistant. During answering the questionnaire, the research assistant was always present, and participants were encouraged to ask him if they had any questions. Later, the research assistant confirmed that none of the questions had been left unanswered. The Health Literacy in Dentistry scale [27] measures a person’s ability to access, process, perceive, and understand the basic oral health-related information required to make informed oral health decisions. Seven conceptual domains are represented by the 14 items in the form: understanding, utilization, access, support, receptivity, economic barriers, and communication. Each item was graded on a 5-point Likert scale ranging from 1 (“no difficulty”) to 5 (“Unable to do”). The possible range of summary scores after re-coding of 5 to 0, 4 to 1, 3 to 2, 2 to 3, and 1 to 4 is 0–56. The possible range of HeLD-14 scores is from 0 (lowest OHL) to 56 (highest OHL). Responses were dichotomized by splitting into low literacy (responses of “with some difficulty” and “very difficult”) and high literacy (“without difficulty” and “little difficulty”) [29]. Health Literacy in Dentistry (HeLD − 14) higher scores indicate a better understanding of oral health. Further, 6 questions of knowledge [28] were presented with a correct answer. Furthermore, the attitude questions [8] were based on a Likert scale ranging from 1 (“strongly agree”) to 5 (“strongly disagree”). The scores were re-coded for 5 to 0, 4 to 1, 3 to 2, 2 to 3 and 1 to 4. The independent variables were age, gender, education, teaching level, income. The dependent or outcome variables were OHL level (low and high), knowledge, and attitude of school teachers.

### Statistical analysis

After collecting the data, it was imported into spss from excel. SPSS software version 26 was used to enter and analyze the data (IBM SPSS, Chicago, IL, USA software version 26.0). Normality assumptions of data was fulfilled by Shapiro-Wilk test. The frequency, and percentage were used to express descriptive statistics. Multiple Logistic regression was applied to check the relationship between OHL and the associated factors. Chi‑square test was applied to evaluate knowledge of study participants. The level of significance was set up at p < 0.05.

## Results

**Fig. 2:**
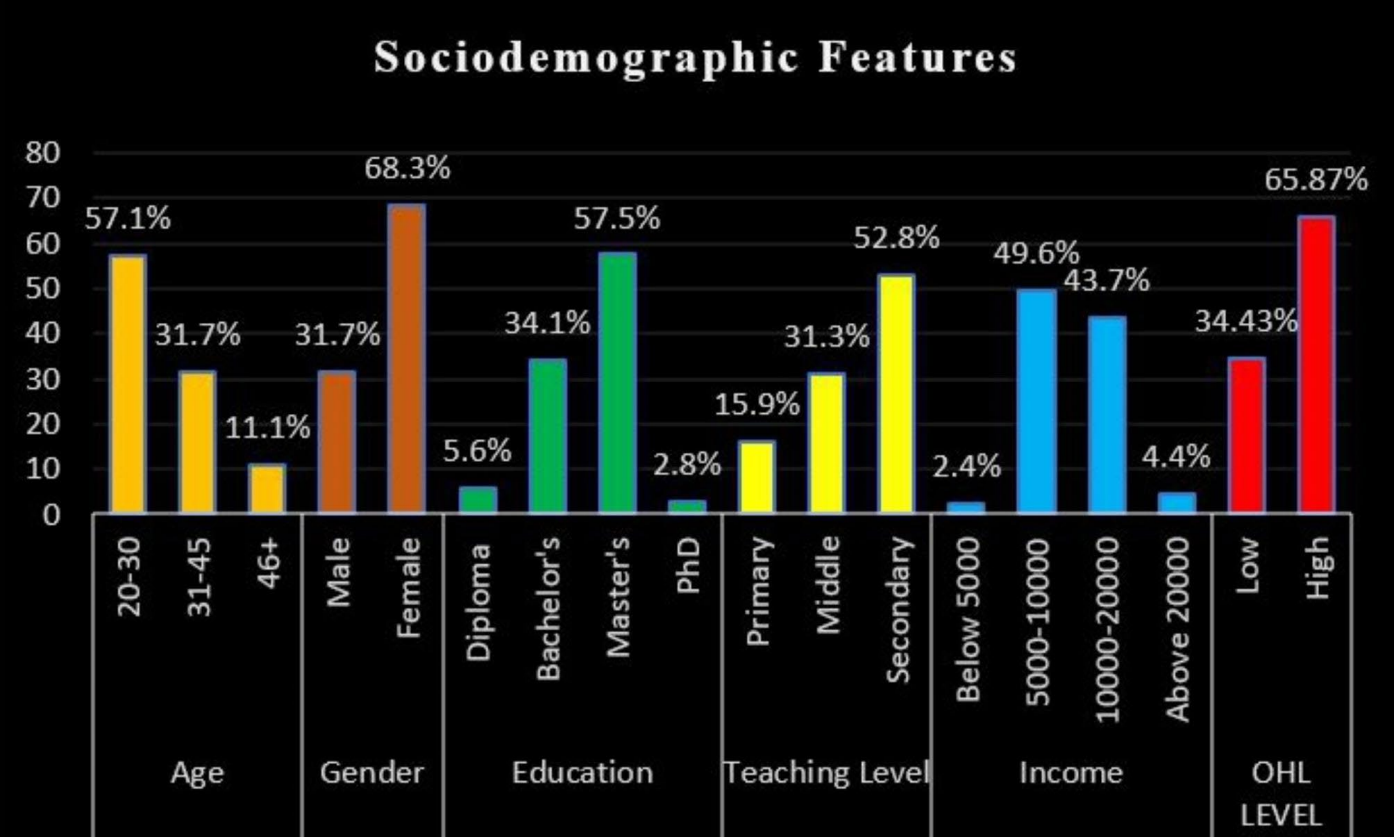
Sociodemographic features

A total of 260 school teachers with a Mean age of school teachers 32.25 ± 8.46 participated in the study, but the response from 8 participants was excluded because they left the questionnaire incomplete and due to missing data. Hence the total participants were 252 (response rate 96.96%). On analysis of the sociodemographic profile of teachers with age, gender, education level, teaching level, and income, it was seen that a majority of the teachers were female (68.3%) with 20–30 years (57.1%) of age group and most of the teachers were having master’s degree (57.5%), teaching in secondary (52.8%) level with 5000–10,000 Riyals (49.6%%) monthly income. The OHL level was high (65.87%) among the school teachers (Fig. 2).

Table 1 demonstrate the multiple logistic regression model showing the association between age, education, and OHL level of school teachers. After adjustment for sociodemographic factors age (OR = 0.219, 95% CI: 0.058–0.834), education (OR = 9.053, 95% CI: 1.135–72,023) were significantly associated with OHL of school teachers. The odds of OHL among school teachers age group 20–30 were higher compare to other age group. The OHL among bachelor’s degree of education of school teachers were having higher odds compare to other education levels. The two-way interaction between all the independent variables were tested one by one, variables with p-value < 0.05 were included in the model. To detect the outlier in multiple logistic regression, the case wise listing of residuals was used. The outliers outside 3 standard deviations were set in this statistical computation. The outlier was listed and excluded from the analysis while fitting the final multiple logistic regression. The multicollinearity was assessed by the correlation matrix between all the variables and by checking the standard error. A good correlation was found between the variables with minimal standard error. The model fitness was assessed by the Hosmer-Lemeshow goodness of fit test with p-value 0.166 which is > 0.05, the discrepancy was minimal between observed and expected probability showing the model is fit.

**Table 1:** Final model of the associated factors for OHL level by multiple logistic regression

Variables	β	SE	Wald	p-value	Adjusted OR	95%CI for Adjusted OR	
						Lower	Upper
Age	-1.517	0.681	4.954	0.026*	0.219	0.058	0.834
Gender	0.311	0.344	0.821	0.365	1.365	0.696	2.678
Education	2.203	1.060	4.323	0.038*	9.053	1.135	72.23
Teaching level	-0.866	0.530	2.792	0.095	0.412	0.146	1.166
Income	-1.440	1.378	1.092	0.296	0.273	0.016	3.528

*Significant p-value < 0.05; Multiple Logistic Regression was applied

The Model reasonably fits well. Model assumptions are met

There is no interaction and multicollinearity problem

Dependent variable: OHL-LEVEL (LOW, HIGH)

β-Beta Coefficient; SE-Standard Error; Wald-Wald Statistics; OR-Odds Ratio; CI-Confidence Interval

The knowledge of school teacher was assessed by questions such as what exactly the dental plaque is, what dental plaque can cause, the bleeding of gums is a sign of, how to avoid periodontal disease, what is dental caries, and when is the best time for tooth brushing. Table 2 shows that 67.0% and 58.3% of school teachers know what dental plaque is and what can cause dental plaque, respectively. further, 71.8% and 85.3% of school teachers know bleeding is a sign of gums disease and how to avoid periodontal disease, respectively. furthermore, 66.3% and 67.1% of school teachers recognize what dental caries is and which is the best time for tooth brushing. Female participants showed better performance with respect to all the knowledge questions, a significantly higher level of knowledge (p-value < 0.05) was reported with all the questions except the second question (dental plaques causes).

**Table 2:** Distribution of schoolteachers who correctly answered the knowledge questions according to gender (*n* = 252)

Questions on Knowledge	Answer Distribution			
	All school Teachers n = 252(%)	Male n = 80(%)	Female n = 172(%)	*p*-value
So, what exactly is dental plaque?				
**They are soft deposit on tooth** They are hard deposit on tooth It is the discoloration of tooth Do not know	**169 (67.0)** 42 30 11	**48 (28.4)** 23 04 05	**121 (71.6)** 19 26 06	0.001*
The dental plaque causes.				
The teeth weakness The discoloration of teeth **The gum disease** Do not know	46 49 **147 (58.3)** 11	17 19 **40 (27.2)** 04	29 29 **107 (72.8)** 07	0.392
The bleeding of gums is a sign of?				
**Gums disease** Healthy gingiva Weakness of periodontium Do not know	**181 (71.8)** 08 39 24	**52 (28.7)** 03 10 15	**129 (71.3)** 05 29 09	0.007*
How to avoid periodontal disease?				
By consuming the soft food **By means of tooth brush and dental floss** By consuming Vit C Do not know	04 **215 (85.3)** 10 23	01 **60 (27.9)** 06 13	03 **155 (72.1)** 04 10	0.008*
What the dental caries is?				
The discoloration of teeth The enamel loss **The tooth destruction** Do not know	40 17 **167 (66.3)** 26	15 08 **41 (24.5)** 15	25 09 **126 (75.5)** 11	0.002*
When is the ideal time for tooth brushing?				
In morning In afternoon **Before sleep** Do not know	73 2 **169 (67.1)** 08	33 0 **45 (26.6)** 02	40 02 **124(73.4)** 06	0.026*

*Significant value < 0.05; Chi-Square Test; All the Correct Answers are Bold

**Fig. 3:**
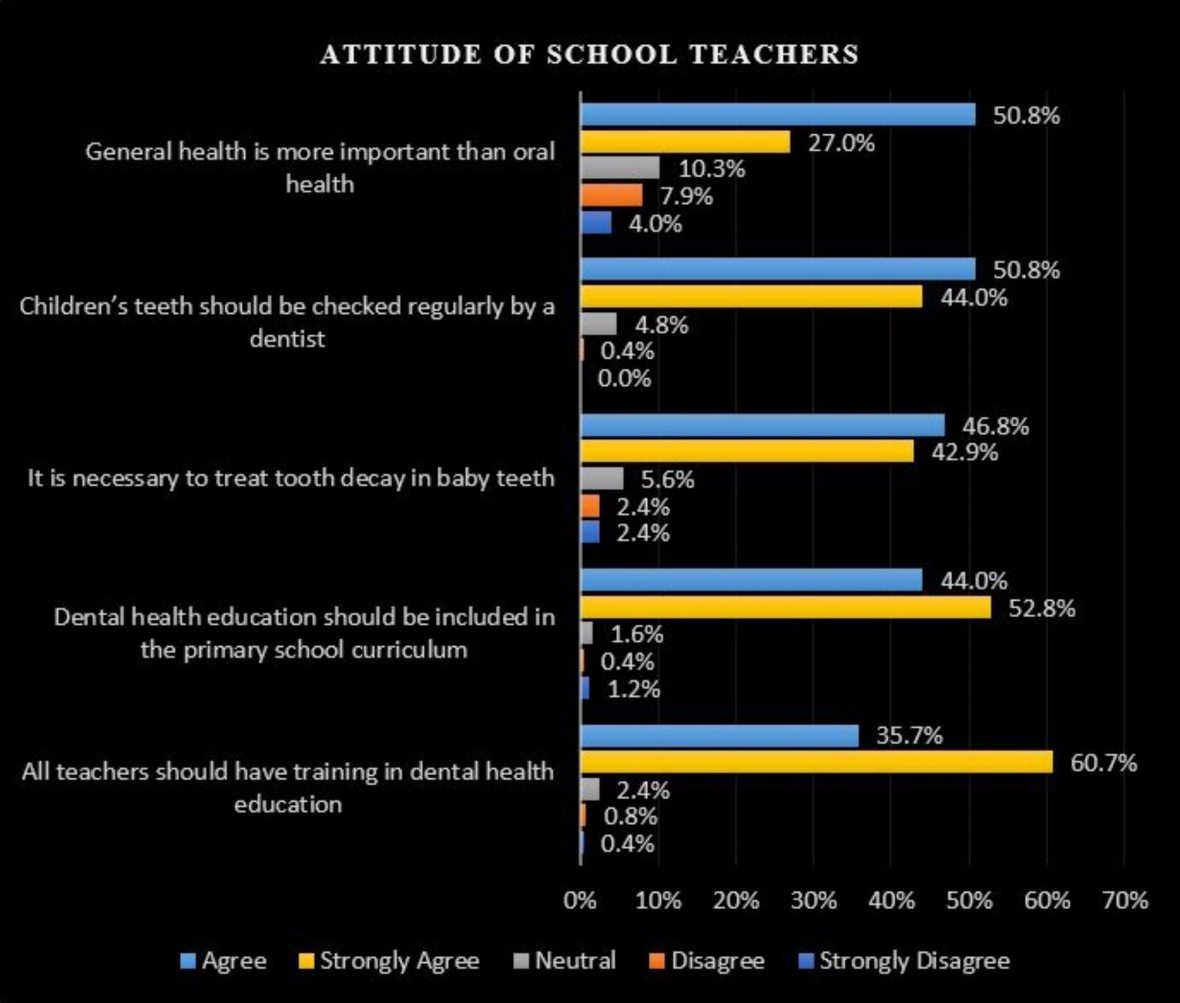
Attitude of schoolteachers regarding oral health and oral health education

Finally, the teachers’ attitudes toward oral health was identified with their levels of oral education. It was revealed that 94.8% of teachers agreed that children’s teeth should be checked by a dentist on a regular basis, while 96.8% agreed that dental health education should be included in the primary school curriculum and that all teachers should receive dental health education training (Fig. 3).

## Discussion

The present study provides a comprehensive view at the knowledge, OHL, and attitude of school teachers with regard to oral health in the city of Najran, Saudi Arabia. The epidemiological data on oral health is limited among school teachers of Saudi Arabia. There are no valid and reliable reports accessible on OHL, knowledge, and attitude among schoolteachers. Therefore, our study provided valid information and helped prepare and implement future school-based oral health education programs. Ramroop et al. [8] demonstrated that, despite teachers’ knowledge about the causes of dental caries and gum diseases in Trinidad, West Indies, significant barriers to the implementation of oral health education by teachers included a lack of resources and inadequate training. Studies conducted in the majority of other developing countries, such as Israel, Romania, and Kuwait, have shown that despite the fact that teachers lacked understanding of oral health, they were enthusiastic about educating their students about oral health [15, 30, 31]. It was also discovered that school teachers lacked the motivation to engage in extracurricular activities that required close supervision and took place during class hours. According to research by Nyandindi et al. [32] even though some teachers were quite knowledgeable about oral health, they were not persuaded to include oral health initiatives.

### OHL among school teachers

Traditionally, studies have concentrated on the level of OHL among patients and a particular group of professionals. In this study, we focused on the OHL of school teachers who unquestionably speak and pronounce better than others. The OHL among the schoolteachers was found to be high (65.87%) with a mean score 39.77 ± 7.94. Similar results were reported among a studies conducted in the Indian population with a mean score 75.83 ± 9.9 [33], and among Iranian population with high OHL 78.8% [34]. According to the multiple regression analysis, school teachers between the ages of 20 and 30 have twice the likelihood of having high OHL than school teachers in other age groups. Findings like these indicate that younger participants were more conscious of their oral health [35]. Our study results revealed a significant association between OHL level and age (p = 0.026*) of the school teachers. Similar findings were reported in studies among Iranian school teachers (r = -0.19; p < 0.001) [34] and Indian school teachers (p < 0.001) [35, 36]. In contrast a study among Indian school teachers reported no significant association between OHL and age [33]. According to the findings of the studies carried out by McQuistan et al. [37] and Haridas et al. [38] an increase in age was found to be associated with a low level of OHL. It is important to note that as people get older, their motivation tends to decrease, while their personal and familial responsibilities tend to increase, which could be the cause of poor OHL among older age groups [34].

Our study results also revealed a statistically significant association between OHL level and education (p = 0.038*) of the school teachers. This finding agrees with the studies conducted among Indian school teachers [33], American participants [39] and Iranian participants [40, 41]. On the other hand, a survey conducted among teachers in Indian schools found no significant association between OHL and education [42]. As a result of advancements in educational standards, there has been a proportional rise in the level of OHL. People with higher education levels are more likely to benefit from educational and counseling programs, and they also have a greater understanding of their education [34]. However, the association between OHL of school teachers with gender, teaching level and income was not found to be statistically significant. According to the findings of our study, both female teachers and male teachers had high levels of OHL. However, the mean level of OHL among female teachers was substantially higher than that of male teachers. Similar findings were reported among studies conducted by Sistani et al. [40] and Ramandeep et al. [43]. This is acceptable, considering the differences in personality traits between both the genders. Women are more likely than males to seek dental care because they use more information resources to maintain their beauty and take care of their children [40]. Previous studies has demonstrated that women have a higher likelihood to participate in oral health programs, and that the gender effect manifested differently in terms of OHL [34, 44]. In contrast, it has been shown that there is no difference in OHL between the sexes in studies conducted in the United States and Canada [45–47], and a study conducted in Korea [48] found that males were having higher OHL than women.

### Knowledge among school teachers

Schoolteachers have long been thought of as potentially powerful primary agents of social development, capable of shaping a student’s future knowledge, attitude, and behavior [15]. Our study results revealed that 67.0% of school teachers answered correctly about the dental plaque and showed a significant association with their gender (p = 0.001). This agrees with the study conducted by Jagan et al. [49]. The female teachers (71.6%) had more knowledge about dentistry than their male (28.4%) counterparts. In this and other communities, it should be noted that most school teachers are female. 58.3% of school teachers know that gum disease is caused by dental plaque. No significant association was reported in relation to gender (p = 0.392) of school teachers. This finding is similar to a study done by Almas et al., [9] among the school teachers of Saudi Arabia.

Further, 71.8% of teachers were aware that bleeding is an indication of gum disease. A significant association was seen concerning their gender (p = 0.007*). The teachers were aware of bleeding gums and the causes of bleeding gums, as well as the fact that if the mouth were washed every day, gum diseases could be avoided. However, 85.3% of school teachers knew how to prevent periodontal disease by using toothbrush and dental floss, a significant association was found in relation to their gender (p = 0.008*). Similar results were found in a study done by Ramroop et al. [8] in which 89.8% of school teachers knew that the regular brushing of teeth could prevent gum disease. 66.3% of school teachers had knowledge of what dental caries is, and a significant association was found in relation to their gender (p = 0.002*). this agrees with the results found in a study done by Shodan et al. [50]. School teachers with high educational level and good working experienced has better knowledge about the dental caries, which is justified as knowledge obtained through their years of teaching experience. Furthermore, 67.1% of school teachers had knowledge about the best time of brushing teeth, that is when done at night time before going to sleep will prevent dental caries. The school teachers who are trained as health promoters can influence the efficacy of health and health literacy knowledge transmission and serve as catalysts for the community in which they are embedded, resulting in the successful health maintenance of the population [51].

### Attitude among schoolteachers

The findings of our study revealed that school teachers have a positive attitude toward oral health. The dental health education should be included in the primary school curriculum, according to 96.8% of teachers, and 96.4% of school teachers reported that all teachers should be trained in dental health education. This is consistent with the findings attained from the studies done by Ramroop et al. 2011 [8]. Dental health education should be promoted through secondary school curriculum in Saudi Arabia, and teachers should be given appropriate training to participate and contribute actively in oral health promotion programs in schools, based on their willingness and interest. As a component of their in-service professional development, the staff should be given access to a comprehensive oral health training programme that is both well-designed and attentive to their needs [52]. It should make it possible for staff members to improve their lifestyles by adopting healthier habits, gain new skills, and incorporate their own knowledge and experience into their teaching. They are able to identify critical policies and practices that promote oral health and overall well-being in school and the community by working with the school health team, parents, and the local community [52]. In summary, the literature has revealed that the intervention programmes produced favorable results, particularly those that included oral health education for children, teachers, and parents, supervised brushing, and the provision of fluoride toothpaste and brushes. The delivery of oral health education to preschoolers through enjoyable activities may also be advantageous. In addition to the school teachers, parental involvement is a factor that plays a role in assessing the effectiveness of the programmes, which may highlight the necessity of providing parents with information about oral health [53].

### Limitations

A small sample size is one of the limitation of our study. The major challenge was gaining access to schools and gaining the approval of school administrators and educational personnel to carry out the research project, which was organized and supported by the education department. Since the oral health-related questions were self-reported, there is the potential for reporting that is not accurate. Further research can be done on a large sample size and also see the association between the oral health knowledge, literacy and attitude of teachers, and oral health status of school children.

## Conclusions

According to the findings of our study it is concluded that overall, school teachers have high OHL, adequate knowledge, and a positive attitude toward oral health. The female teachers had more knowledge about dentistry than their male counterparts. Further research is advised in order to assess and compare oral health status of school teachers with OHL, knowledge, and attitudes, and determine whether they provide oral health education to school children. It is recommended that by implementing teacher training programmes that improve oral health knowledge, literacy and attitude, and approaches to dental health education within a school setting, collaboration between institutions associated with school health, the dental profession, and those working to develop school curricula could allow school teachers to play a significant role in oral health promotion for young children.

## Data Availability

The datasets used and/or analysed during the current study are available from the corresponding author on reasonable request.

## Abbreviations

OHL: Oral Health Literacy
HeLD: Health Literacy in Dentistry
WHO: World Health Organization
